# Real-Life Patterns of Exacerbations While on Inhaled Corticosteroids and Long-Acting Beta Agonists for Asthma over 15 Years

**DOI:** 10.3390/jcm9030819

**Published:** 2020-03-18

**Authors:** Michael J. McGeachie, Alberta L. Wang, Sharon M. Lutz, Joanne E. Sordillo, Scott T. Weiss, Kelan G. Tantisira, Carlos Iribarren, Meng X. Lu, Ann Chen Wu

**Affiliations:** 1 Channing Division of Network Medicine, Brigham and Women’s Hospital, Boston, MA 02115, USA; michael.mcgeachie@channing.harvard.edu (M.J.M.);; 2 PRecisiOn Medicine Translational Research (PROMoTeR) Center, Department of Population Medicine, Harvard Pilgrim Health Care Institute and Harvard Medical School, Boston, MA 02115, USA; 3 Kaiser Permanente Division of Research, Kaiser Permanente, Oakland, CA 94612, USA

**Keywords:** asthma, inhaled corticosteroids, long-acting beta agonist, clinical data, exacerbations, efficacy

## Abstract

Asthma affects more than 300 million people in the world, costs over $80 billion annually in the United States, and is efficaciously treated with inhaled corticosteroids (ICS). To our knowledge, no studies have examined the real-world effectiveness of ICS, including the combination therapy consisting of ICS and long-acting beta agonists (LABAs), and patterns of use over a 15-year time period. We used data from the Kaiser Permanente Northern California multi-ethnic Genetic Epidemiology Research on Adult Health and Aging (GERA) Cohort which comprises longitudinal electronic health record data of over 100,000 people. Data included longitudinal asthma-related events, such as ambulatory office visits, hospitalizations, emergency department (ED) visits, and fills of ICS and ICS–LABA combination. Asthma exacerbations were defined as an asthma-related ED visit, hospitalization, or oral corticosteroid (OCS) burst. We used an expected-value approach to determine ICS and ICS–LABA coverage over exacerbation events. We compared rates of exacerbation of subjects on ICS or ICS–LABAs to their own rates of exacerbation when off controller medications. We found ICS–LABA therapy had significant effects, reducing all types of exacerbations per day by a factor of 1.76 (95% CI (1.06, 2.93), *p* = 0.03) and, specifically, bursts per day by a factor of 1.91 (95% CI (1.04, 3.53), *p* = 0.037). In conclusion, ICS–LABA therapy was significantly associated with fewer asthma-related exacerbations in a large population of individuals with asthma who were followed for 15 years.

## 1. Introduction

Asthma is the most common chronic illness in childhood, costs over $80 billion annually in the United States [1], and is efficaciously treated with inhaled corticosteroids (ICS) [2]. When patients are not adequately controlled by ICS, randomized controlled trials have established that ICS used in combination with long-acting beta agonists (LABAs) are superior to increasing dosage of ICS alone [3].

Observational studies of ICS and ICS–LABA combinations have established effectiveness in reducing serious complications of asthma: hospitalizations, emergency department visits, and short-term supplemental courses of systemic steroids, collectively termed exacerbations [4,5]. A large study of a two-year period found that current (but not history of) adherence to ICS treatments was associated with reduced asthma exacerbations [6]. Others found that ICS improved asthma control and lung function, but only in patients with eosinophilia [7].

Further, observational studies have established the efficacy and effectiveness of ICS combined with LABA, either concurrently or in a combined medication [8,9]. Many have studied ICS–LABAs effectiveness in the treatment of chronic obstructive pulmonary disease (COPD) [10,11,12]. There have been fewer observational studies of treatment of asthma, although these have established effectiveness of ICS–LABAs, demonstrating fewer exacerbations [13].

However, these studies considered modest follow-up periods, one year at the most. To our knowledge, no studies have examined the real-world effectiveness of ICS, including ICS and LABA combination therapy, and patterns of use over a 15-year time period. The objective of this study was to evaluate health care utilization events among adults with asthma after initiation of ICS. 

## 2. Methods

We used data from the Kaiser Permanente Northern California multi-ethnic Genetic Epidemiology Research on Adult Health and Aging (GERA) Cohort [14] that comprises longitudinal electronic health record data on over 100,000 people. Data included longitudinal asthma-related event data, where “events” were dates of ambulatory office visits, hospitalizations, emergency department (ED) visits, and fills of ICS and ICS–LABA combination.

**Figure 1:**
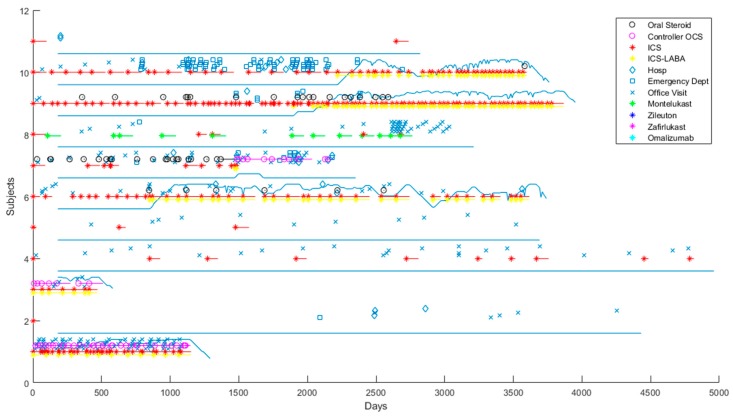
Random Genetic Epidemiology Research on Adult Health and Aging (GERA) patients. Average number of days of inhaled corticosteroids (ICS)–long-acting beta agonists (LABAs) is shown. Each patient is represented by a horizontal track, with a solid line indicating ICS–LABA coverage, and discrete events and prescription fills represented by shapes. Open shapes represent exacerbations: black circles are oral steroid bursts, diamonds are asthma-related hospitalizations, open squares are asthma-related emergency department visits. Colored stars represent prescription fills for a controller medication, with a horizontal line of the same color indicating the duration of that prescription. Chronic oral corticosteroids (OCS) are treated as a controller medication and represented by pink circles. The solid blue line indicates the percentage coverage of ICS–LABA, with a low line indicating 0% coverage, and a high line indicating 100% coverage. We averaged the duration of each ICS–LABA prescription over 180 days to quantify the uncertainty of adherence to ICS–LABAs.

Asthma exacerbations were defined as an asthma-related ED visit, hospitalization, or oral corticosteroid (OCS) burst. OCS bursts were defined as single OCS prescriptions administered as a short-term “burst” to treat a sudden worsening of asthma symptoms, thus excluding long-term OCS use as a controller medication. Figure 1 shows several random patients from GERA.

Subjects with asthma-related events were analyzed for drug response, using the rates of exacerbations on and off of each controller medication (ICS and ICS–LABAs). We a priori filtered out patients with missing dates, without events, and no events after their first ICS prescription. To avoid bias due to outliers, we limited each patient’s longitudinal data to 15 years. To limit confounding [15], we limited attention to subjects with exacerbations both on and off of each particular asthma controller medication, and we considered only the data of subjects from the start of their first ICS prescription.

Duration of ICS use was computed using an expected-value approach, where the subject was considered to be treated with the controller in proportion to the length of time covered by prescriptions after medication fills within a window size of 180 days. This method allowed us to statistically account for the uncertainty in medication adherence by considering each event to be covered by the controller in proportion to the number of covered days within the preceding 180 days. For example, a hospital admission for asthma occurring after a period where the patient was covered by ICS for 60 of 180 days was considered to be one-third of an exacerbation on ICS and simultaneously two-thirds of an exacerbation off ICS. OCS events with greater than 50% OCS coverage were not considered to be bursts, but rather use of OCS as a long-term controller medication.

For each subject, we computed the exacerbation rate on ICS and ICS–LABAs and compared it to the subject’s exacerbation rate when not taking any controller medications. The rates were log-transformed, while adding a small constant to avoid logarithms of zero (0.00001). Associations were conducted by linear regression while controlling for age, height, weight, sex, and smoking history. Patients with missing values for these covariates were imputed with the mean value, which biased toward the null result of no association. All computations were performed in MATLAB R2018a (The Mathworks, Natick, MA, USA). 

## 3. Results

Our main analysis that focused on ICS response had 4137 subjects who had a diagnosis of asthma, fill of ICS, at least 365 days of observation, and with more than 360 days of ICS treatment. We also considered otherwise similar subjects with more than 360 days of ICS–LABA treatment (n = 1844). When the ICS–LABA retained group was compared with subjects who were excluded, the retained subjects were more likely to be male, former smokers, and white and less likely to be Asian (Table 1). 

To improve our ability to assess subjects’ response to ICS and ICS–LABA therapy, we compared a subjects’ rate of exacerbation on ICS or ICS–LABAs to her/his rate off of any therapy. We did not find significant effects for ICS monotherapy. When we limited the analysis to 1295 subjects who had asthma-related exacerbations both on ICS–LABAs and off controller medications (Table 2), we found ICS–LABAs had significant effects, reducing all types of exacerbations per day by a factor of 1.76 (95% CI (1.06, 2.93), *p* = 0.03) and reducing specifically OCS bursts per day by a factor of 1.91 (95% CI (1.04, 3.53), *p* = 0.037).

When analysis was stratified by race or smoking status, significant effects were not observed.

## 4. Discussion

Our study has several key findings. First, ICS–LABA combined therapy is associated with decreased exacerbations from asthma, including asthma-related ED visits, hospitalizations, or OCS bursts in a large, real-life population of subjects with asthma followed for 15 years. Furthermore, we found that ICS–LABA therapy is associated with decreased OCS bursts alone.

The strengths of our study include a large and diverse population with a 15-year follow-up period, which increases the generalizability of our results. Similarly, we performed an analysis of rates of exacerbations using more of the available data rather than the time to first event, as has been common practice in the literature [16,17]. Patients were used as their own controls to limit confounding by indication, as done in some previous work [4], although over long periods of observation a person’s general disease severity may have worsened.

Adherence to medication and measuring adherence to medication are typically among the most difficult issues in observational studies of comparative effectiveness. In fact, there has been a number of studies showing that adherence to ICS–LABAs is increased when the medications are combined into a single dose, rather than administered as two separate medications [18,19]. We addressed adherence here by using an expected-value-based approach to assigning exacerbations to periods of presumed greater or lesser adherence. Some previous work has estimated adherence similarly, but then chose to dichotomize adherence on the basis of a threshold (0.8) [19] rather than quantify the uncertainty as we did. Our method also blurs the boundaries between baseline (before advent of medication) and follow-up (after advent of medication) periods of observation [20], allowing us to use patients’ entire histories to increase power. 

The limitations of our study include the possibility of unadjusted confounding by indication, although the restrictions we made on the subjects included were aimed at obtaining a subcohort with the best possible phenotype validity [21]. Although our results as presented were adjusted for race and smoking status, we did not have significant numbers of non-white participants or of current smokers to investigate stratified effects of ICS and ICS–LABAs.

In conclusion, ICS–LABA therapy was significantly associated with reduced asthma-related exacerbations in a large population of individuals with asthma over 15 years. 

## Figures and Tables

**Table 1:** Comparison of patients excluded vs. retained in the ICS–LABA analysis. Exacs: exacerbations.

	Retained	Excluded	*p*-Value
N	1844	11,917	
Age (years)	64.00 (+/−11.37)	63.66 (+/−13.21)	0.29
Weight (kg)	80.9 (+/−19.2)	80.0 (+/−19.2)	0.065
Smoking			
Never	796 (43.17%)	6326 (53.08%)	0.23
Former	851 (46.15%)	4578 (38.42%)	0.019
Current	113 (6.13%)	529 (4.44%)	1.30 × 10^−13^
Height (in)	65.76 (+/−23.62)	63.98 (+/−45.22)	0.1
Gender (n Male)	696 (37.74%)	3879 (32.55%)	1.10 × 10^−5^
Race/ethnicity			
Asian	111 (6.02%)	883 (7.41%)	0.032
Black	83 (4.50%)	467 (3.92%)	0.23
Hispanic	127 (6.89%)	1013 (8.50%)	0.019
Other	15 (0.81%)	88 (0.74%)	0.73
White	1508 (81.78%)	9466 (79.43%)	0.02
Total Days Observed	3391 (+/−866)	2405 (+/−1277)	3.20 × 10^−217^
Total Days Not Covered	1543 (+/−808)	1736 (+/−1137)	2.10 × 10^−12^
Total Bursts	7.67 (+/−9.86)	2.56 (+/−5.74)	5.40 × 10^−213^
Total Hospitalizations	1.57 (+/−2.78)	0.80 (+/−1.94)	4.20 × 10^−49^
Total ED visits	2.21 (+/−4.10)	1.12 (+/−3.28)	4.20 × 10^−37^
Days covered by OCS	0.05 (+/−0.11)	0.03 (+/−0.10)	3.30 × 10^−13^
Days covered by ICS	0.15 (+/−0.17)	0.20 (+/−0.23)	7.80 × 10^−19^
Days covered by Montelukast	0.10 (+/−0.20)	0.06 (+/−0.18)	3.30 × 10^−24^
Days covered by ICS–LABA combination	0.37 (+/−0.20)	0.03 (+/−0.13)	0
Total Exacs on ICS	1.83 (+/−3.73)	0.99 (+/−2.45)	2.10 × 10^−36^
Total Exacs on Montelukast	1.38 (+/−4.06)	0.34 (+/−2.13)	4.30 × 10^−62^
Total Exacs on ICS–LABAs	4.04 (+/−5.56)	0.27 (+/−1.95)	0
Bursts covered by ICS	1.22 (+/−2.47)	0.54 (+/−1.55)	1.20 × 10^−56^
Bursts covered by Montelukast	0.90 (+/−2.72)	0.20 (+/−1.25)	4.00 × 10^−73^
Bursts covered by ICS–LABAs	2.54 (+/−3.68)	0.16 (+/−1.16)	0
ED/Hosps covered by ICS	0.61 (+/−1.90)	0.45 (+/−1.36)	8.40 × 10^−6^
ED/Hosps covered by Montelukast	0.48 (+/−1.90)	0.14 (+/−1.19)	1.00 × 10^−24^
ED/Hosps covered by ICS–LABAs	1.50 (+/−2.90)	0.12 (+/−1.05)	5.20 × 10^−303^
ED/Hosps covered by OCS	0.41 (+/−1.84)	0.13 (+/−1.69)	1.40 × 10^−10^

**Table 2:** Comparison of subjects with and without exacerbations (both on ICS–LABAs and off treatment). ED: emergency department, Hosps: hospitalizations, Exacs: exacerbations of all types.

	No Exacerbations on ICS/LABAs	Exacerbations on ICS/LABAs	*p*-Value
N	549	1295	
Age (years)	62.29 (+/−11.85)	64.73 (+/−11.09)	2.30 × 10^−5^
Weight (kg)	78.77 (+/−18.3)	81.87 (+/−19.6)	0.0018
Smoking			
Never	238 (43.35%)	558 (43.09%)	0.92
Former	254 (46.27%)	597 (46.10%)	0.95
Current	38 (6.92%)	75 (5.79%)	0.35
Height (in)	66.29 (+/−3.84)	65.54 (+/−28.07)	0.54
Gender (n Male)	196 (35.70%)	500 (38.61%)	0.24
Race/ethnicity			
White	459 (83.61%)	1049 (81.00%)	0.19
Asian	31 (5.65%)	80 (6.18%)	0.66
Black	21 (3.83%)	62 (4.79%)	0.36
Hispanic	33 (6.01%)	94 (7.26%)	0.33
Other	5 (0.91%)	10 (0.77%)	0.76
Total Days Observed	3327.22 (+/−874.55)	3419.35 (+/−861.36)	0.037
Total Days Not Covered	1374.14 (+/−784.18)	1615.23 (+/−807.89)	4.10 × 10^−9^
Total Bursts	1.97 (+/−3.58)	10.09 (+/−10.65)	3.20 × 10^−63^
Total Hospitalizations	0.42 (+/−0.85)	2.06 (+/−3.14)	4.10 × 10^−32^
Total ED visits	0.60 (+/−1.12)	2.89 (+/−4.67)	5.90 × 10^−29^
Days covered by OCS	0.01 (+/−0.04)	0.06 (+/−0.12)	1.00 × 10^−21^
Days covered by ICS	0.16 (+/−0.19)	0.14 (+/−0.16)	0.0028
Days covered by Montelukast	0.11 (+/−0.22)	0.10 (+/−0.19)	0.37
Days covered by ICS–LABA combination	0.39 (+/−0.22)	0.36 (+/−0.19)	0.0015
Total Exacs on ICS	0.74 (+/−1.69)	2.29 (+/−4.23)	2.40 × 10^−16^
Total Exacs on Montelukast	0.53 (+/−1.92)	1.74 (+/−4.63)	4.50 × 10^−9^
Total Exacs on ICS–LABAs	1.25 (+/−2.38)	5.22 (+/−6.07)	3.50 × 10^−47^
Bursts covered by ICS	0.50 (+/−1.34)	1.53 (+/−2.77)	2.10 × 10^−16^
Bursts covered by Montelukast	0.36 (+/−1.46)	1.12 (+/−3.08)	3.40 × 10^−8^
Bursts covered by ICS–LABAs	0.78 (+/−1.72)	3.28 (+/−4.02)	1.30 × 10^−42^
ED/Hosps covered by ICS	0.25 (+/−0.71)	0.76 (+/−2.21)	8.40 × 10^−8^
ED/Hosps covered by Montelukast	0.17 (+/−0.73)	0.61 (+/−2.21)	4.20 × 10^−6^
ED/Hosps covered by ICS–LABAs	0.47 (+/−1.04)	1.94 (+/−3.30)	6.80 × 10^−24^
ED/Hosps covered by OCS	0.03 (+/−0.19)	0.57 (+/−2.17)	6.40 × 10^−9^
